# Patellar Tendon Strain Associates to Tendon Structural Abnormalities in Adolescent Athletes

**DOI:** 10.3389/fphys.2019.00963

**Published:** 2019-08-02

**Authors:** Falk Mersmann, Nikolaos Pentidis, Meng-Shiuan Tsai, Arno Schroll, Adamantios Arampatzis

**Affiliations:** ^1^Department of Training and Movement Sciences, Humboldt-Universität zu Berlin, Berlin, Germany; ^2^Berlin School of Movement Science, Humboldt-Universität zu Berlin, Berlin, Germany

**Keywords:** tendon injury, overload, micromorphology, imbalance, youth athletes

## Abstract

High mechanical strain is thought to be one of the main factors for the risk of tendon injury, as it determines the mechanical demand placed upon the tendon by the working muscle. The present study investigates the association of tendon mechanical properties including force, stress and strain, and measures of tendon micromorphology and neovascularization, which are thought to be indicative of tendinopathy in an adolescent high-risk group for overuse injury. In 16 adolescent elite basketball athletes (14–15 years of age) we determined the mechanical properties of the patellar tendon by combining inverse dynamics with magnetic resonance and ultrasound imaging. Tendon micromorphology was determined based on a spatial frequency analysis of sagittal plane ultrasound images and neovascularization was quantified as color Doppler area. There was a significant inverse relationship between tendon strain and peak spatial frequency (PSF) in the proximal tendon region (*r* = −0.652, *p* = 0.006), indicating locally disorganized collagen fascicles in tendons that are subjected to high strain. No such associations were present at the distal tendon site and no significant correlations were observed between tendon force or stress and tendon PSF as well as between tendon loading and vascularity. Our results suggest that high levels of tendon strain might associate to a micromorphological deterioration of the collagenous network in the proximal patellar tendon, which is also the most frequent site affected by tendinopathy. Neovascularization of the tendon on the other hand seems not to be directly related to the magnitude of tendon loading and might be a physiological response to a high frequency of training in this group. Those findings have important implications for our understanding of the etiology of tendinopathy and for the development of diagnostical tools for the assessment of injury risk.

## Introduction

The human muscle-tendon unit is highly adaptive to its mechanical environment. In response to increased mechanical loading, the strength capacity of a muscle may change due to an increased activation level, hypertrophy and/or specific tension (Narici et al., 1989; Aagaard et al., 2002; Erskine et al., 2010). Tendons transmit the forces generated by the muscle to the skeleton and function as biological springs. Due to their elasticity, tendons are able to store and release mechanical strain energy and provide favorable operating conditions for the muscle considering the force-length-velocity relationship of the muscle fibers (Kawakami and Fukunaga, 2006; Lichtwark and Wilson, 2007; Bohm et al., 2018). They are also able to adapt to loading by increasing their stiffness based on changes of their material properties and/or (in the long-term) radial growth (Bohm et al., 2015, for review). A balanced development of muscle strength and tendon stiffness is not only necessary to maintain the operating conditions of muscle fascicles in an optimal range, but also prevent damage to the tendinous tissue that is now subject to higher muscular forces, as the maximum tolerable strain of tendons is quite constant (LaCroix et al., 2013; Shepherd and Screen, 2013). An imbalanced adaptation of muscle and tendon could, therefore, pose a risk of overload injury (Mersmann et al., 2017a). However, muscle and tendon might not necessarily adapt in a balanced manner. The rate of tissue renewal is markedly lower in tendons (Heinemeier et al., 2013b), which might cause a delayed adaptive response with regard to the muscle (Kubo et al., 2010, 2012). Further, the mechanical stimuli that efficiently promote tissue adaptation show differences between muscle and tendon (Arampatzis et al., 2007a; Heinemeier et al., 2013a). During growth, processes of maturation could additionally challenge the development within the muscle-tendon unit (Neugebauer and Hawkins, 2012; Mersmann et al., 2017a). In fact, recent investigations on adolescent volleyball athletes, which is a high-risk group for the development of tendinopathy, provided evidence that an imbalance in the development of muscle and tendon results in an increase of *in vivo* strain of the patellar tendon during maximum voluntary muscle contractions (Mersmann et al., 2014, 2016, 2017b). An increase of tendon strain during maximum effort muscle activity implies an increased mechanical demand for the tendon. It has been shown in cadaveric experiments on human Achilles tendons that time until failure in cyclic loading directly depends on the initial strain induced by the applied load (Wren et al., 2003). Further, there is now growing evidence available that, at least in the Achilles tendon, patients with tendinopathy demonstrate higher levels of strain during contractions (Obst et al., 2018). However, the implications of the increased mechanical demand for the patellar tendon, as observed in adolescent athletes, for the risk of tendon injury so far remain an assumption.

Tendinopathy is frequently associated with tendinosis, which describes degenerative processes and abnormalities within the tendon matrix, including disorganization of the collagenous network, vascular infiltration, increased cellularity and ground substance (Khan et al., 1996; Abat et al., 2017). Some of the consequences of these changes can be evaluated using ultrasound imaging. The assessment of tendon thickening, hypoechogenicity, and vascularity are now established tools for confirming the diagnosis of tendinosis in the clinical setting (Campbell and Grainger, 2001; Warden et al., 2006) and show high reliability (Black et al., 2004; Cook et al., 2005). It even seems that structural abnormalities in asymptomatic tendons can predict the development of future symptoms (in terms of occurrence, not severity), with athletes that show indications for tissue degeneration in the ultrasound examination of the patellar tendon are about fourfold more likely to become symptomatic (McAuliffe et al., 2016). Analysis algorithms for ultrasound image post-processing further enable the quantification of the packing density and orientation of collagen bundles and provide a possibility to non-invasively estimate the structural integrity of the tissue (Bashford et al., 2008; van Schie et al., 2010). With this approach it has been demonstrated that not only is the collagenous scaffold of the tendon disorganized in patients with tendinopathy (Kulig et al., 2013), but the level of disorganization predicts to some extent the mechanical properties of tendons (Kulig et al., 2016). Therefore, it seems well-possible that in individuals with a high risk for tendinopathy, an increased mechanical demand for the tendon (i.e., strain) is associated to structural precursors of the pathology. Since progressive damage to collagen fibrils and fibers reduces the intrinsic extracellular matrix tension and leads to a deterioration of the tendon mechanical properties upon strain-induced overload (Fung et al., 2009; Pingel et al., 2014), estimates of tendon micromorphology might further predict the tendon elastic modulus (Kulig et al., 2016).

Considering the increased levels of patellar tendon strain that have been observed in adolescent athletes at risk of tendinopathy and the structural changes that have been reported to predict tendinopathy, the present study aims to investigate if there is an association between the mechanical and structural characteristics of the patellar tendon that particular group. We hypothesized that while tendon force and stress would not show a direct association to structural abnormalities, the strain as a measure of tendon mechanical demand would predict the structural integrity and occurrence of neovascularization in the patellar tendon. Moreover, we expected the material properties of the patellar tendon to associate with its micromorphology.

## Materials and Methods

### Participants and Experimental Design

Sixteen male adolescent elite basketball players were recruited for the present study. Inclusion criteria were an age of 14 or 15 years, regular participation in basketball training at least four times a week and no neurological or musculoskeletal impairments relevant for the purpose of the study. Mean ± standard deviation of age, body height and mass were 14.8 ± 0.5 years, 185.1 ± 8.2 cm and 72.4 ± 9.4 kg, respectively. Maturity was predicted using age and sitting height (90.1 ± 4.8 cm) in the recalibrated prediction equation for boys suggested by Moore et al. (2015), yielding an estimated average offset from the peak height velocity (PHV) of 0.9 ± 0.7 years. Most players were asymptomatic, yet we included six participants that reported patellar tendon pain during sportive and/or everyday activity, which was assessed using the VISA-P questionnaire (Visentini et al., 1998). However, none of them matched the exclusion criteria that they would not be able to exert maximum isometric voluntary contractions due to pain. One participant reported a history of Osgood–Schlatter disease, yet he experienced no symptoms at the distal attachment in the last 3 month and no signs of structural abnormalities were observed in the ultrasound examination. The participants and legal guardians gave written informed consent to the experimental procedures, which were approved by the ethics committee of the Humboldt-Universität zu Berlin and carried out in accordance with the declaration of Helsinki. All measurements were performed on the dominant leg, which was determined by asking for which leg would be used for kicking a ball. The measurements were done on two separate days, with the magnetic resonance imaging session scheduled not more than 1 week before or after the ultrasound/dynamometry assessment. The assessment of tendon micromorphology and vascularity was performed prior to the one of tendon mechanical properties to avoid acute loading-related responses.

### Assessment of Tendon Morphology and Moment Arm

**FIGURE 1:**
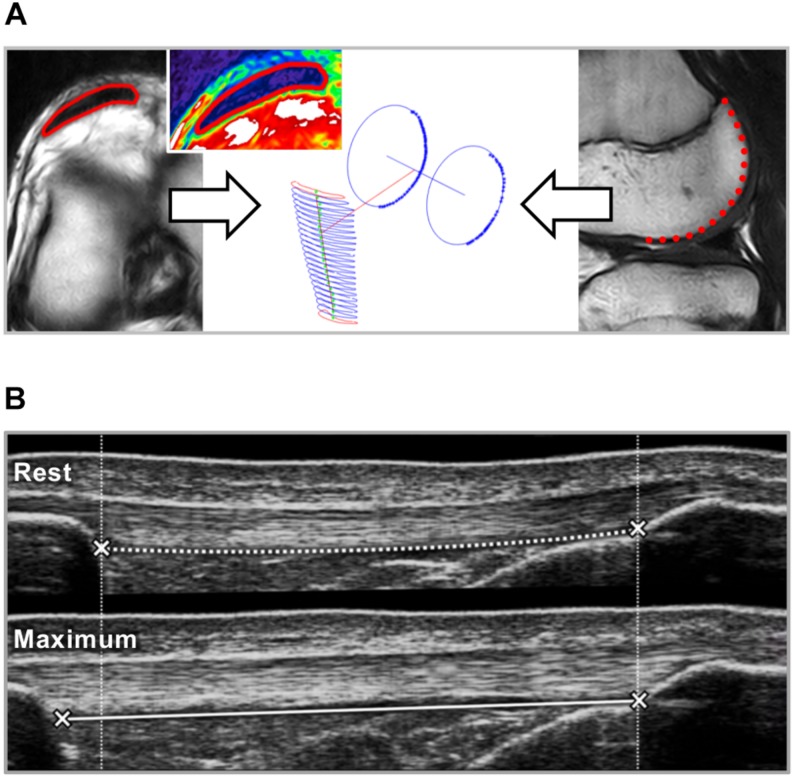
**(A)** Patellar tendon cross-sectional area (CSA) and moment arm were determined based on magnetic resonance images. In transverse plane images the tendon CSA (red) was segmented over the full length of the tendon (left; inset picture shows the tendon in NIH color scale). Sagittal plane segmentations of the posterior contours of the lateral and medial femur condyles were approximated using a circular fit and the line connecting the centers of the circles was defined as rotation axis (Churchill et al., 1998). The perpendicular distance to the tendon line of action represents the tendon moment arm. **(B)** The elongation of the patellar tendon during isometric ramp contractions from rest to maximum was measured using ultrasound. Tendon strain, stiffness and elastic modulus were calculated based on the force-elongation and stress-strain relationship between 50 and 80% of the maximum tendon force or stress.

To determine the cross-sectional area (CSA) and moment arm of the patellar tendon, magnetic resonance images (MRIs) of the participants were captured while lying supine with the dominant knee flexed to 10° (0° = full extension) in a 0.25 T MRI scanner (G-Scan, Esaote, Italy). The knee angle was chosen to reduce the slack of the tendon, which facilitates the subsequent tendon segmentation. In transverse MRI sequences [3D HYCE (GR), 10 ms repetition time, 5 ms excitation time, 80° flip angle, 3 mm slice thickness, one excitation] the boundaries of the patellar tendon were segmented in OsiriX (Version 7.0.2, Pixmeo SARL, Bernex, Switzerland) between the distal apex of the patellar and deep insertion at the tibial tuberosity. As recommended by Couppé et al. (2013), we used the NIH color scale during the segmentation to increase the accuracy of tracing the contours of the tendon. Since it is barely possible to perfectly align the longitudinal axis of the tendon with the longitudinal axis of the MRI scanner, simple transversal plane segmentations would lead to an overestimation of the CSA. Therefore, the digitized patellar tendon CSAs were transformed orthogonal to the line of action of the patellar tendon, which was defined as the line of best fit through the geometrical centers of the respective CSAs (Figure 1A).

For the assessment of the tendon moment arm, we tracked the contours of the posterior part of the femur condyles in sagittal plane sequences of a similar set 3D HYCE scan protocol. A least-squares circular fit was applied to the segmentation and the line connecting the centers of the circle representing the medial and lateral condyle respectively was the approximated rotation axis of the knee (Churchill et al., 1998). The perpendicular distance from the patellar tendon line of action to the axis of rotation represented the patellar tendon moment arm (with regard to the measurement-position of 10° knee flexion).

### Assessment of Tendon Mechanical Properties

The mechanical properties of the patellar tendon were determined based on its force-elongation relationship, which was assessed for isometric contractions by combining inverse dynamics, electromyography and ultrasound imaging. For the kinematic recordings with a motion capture system (Nexus version 1.7.1; Vicon Motion Systems, Oxford, United Kingdom), integrating eight cameras (6x F20, 2x T20) operating at 250 Hz, five reflective markers were fixed to the lateral and medial malleolus, femoral epicondyles and the greater trochanter. Further, for estimating the contribution of the antagonistic muscles during the isometric contractions (Mademli et al., 2004), two bipolar surface electrodes (Blue Sensor N, Ambu GmbH, Bad Nauheim, Germany) were fixed over the mid-portion of the muscle belly of the lateral head of the biceps femoris with an inter-electrode distance of 2 cm after shaving and cleaning the skin. The electromyographic (EMG) activity of the lateral head of the biceps femoris was recorded wirelessly (Myon m320RX, Myon AG, Baar, Switzerland) and integrated into the Vicon system.

Following a standardized warm-up including 10 submaximal isometric contractions as familiarization with the dynamometer (Biodex System III, Biodex Medical, Inc., Shirley, NY, United States), the participants performed three trials of isometric maximum voluntary knee extension contractions (iMVC) at 85° trunk flexion (supine = 0°) and resting knee joint angles of 65 to 75° based on the dynamometer feedback (0° = full extension) in 5° intervals. These resting angles were chosen as, in our experience, the optimum knee angle for force production is usually reached during contraction from a rest position within this range. Subsequently, a 10 cm probe of the ultrasound system (LA923, 7.5 MHz) was fixed in alignment with the longitudinal axis of the patellar tendon with a modified knee brace. The tendon elongation was captured during five trials of isometric ramp contractions (i.e., steadily increasing effort from rest to 90% of the iMVC in about 5 s). The joint angle was the one in which the highest individual iMVC was achieved, to account for the interindividual variation in optimum angle. To be able to account for moments of gravity in the inverse dynamics approach applied to calculate joint moments (Arampatzis et al., 2004) an additionally knee extension trial (driven by the dynamometer at 5°/s) was recorded. Finally, two trials of knee flexion contractions were recorded and to establish an activation-flexion moment relationship that was used to estimate the knee flexion moments generated during isometric contractions by the antagonists when calculating the knee extension moments (Mademli et al., 2004).

To calculate patellar tendon forces, the knee extension moments were divided by the tendon moment arm, which was adjusted to the respective knee joint angle based on the data reported by Herzog and Read (1993). The maximum tendon force (TF_max_) refers to the force calculated for the maximum iMVC trial of each participant. The elongation of the tendon during the ramp contractions was determined by tracking the displacement of the deep insertion of the tendon at the patella and tibial tuberosity using a semi-automatic software (Tracker Video Analysis and Modeling Tool V. 5.06, Open Source Physics, Aptos, CA, United States; see Figure 1B for a schematic illustration). The force-elongation relationship of the five trials of each participant was averaged to achieve an excellent reliability (Schulze et al., 2012) and calculated up to 80% TF_max_ to account for interindividual differences in the capability to exert force during the ramp contraction relative to their maximum (Mersmann et al., 2017b, 2018). This 80% TF_max_ was the highest common force relative to the individual maximum achieved by all participants. Tendon stiffness was calculated as slope of a linear regression between 50 and 80% TF_max_. Tendon stress was calculated for the proximal and distal 40% of tendon length by dividing TF_max_ by the average tendon CSA of the respective section. Tendon elastic modulus was calculated as slope of a linear regression of the stress-strain relationship between 50 and 80% of maximum tendon stress, which was in this case calculated using the average CSA of the full tendon.

### Assessment of Tendon Micromorphology

Tendon micromorphology was assessed based on a spatial frequency analysis of ultrasound images obtained at the proximal and distal part of the patellar tendon, respectively. The participants were positioned supine with the dominant leg flexed in the knee joint to 90° (0° = full extension), which was measured based on kinematic data captured with the Vicon system. The joint angle was chosen as, based on our experience, the tendon slack is removed and fascicles straightened, yet the passive joint forces are estimated to be as low as not to induce substantial strain (O’Brien et al., 2010). The linear transducer of an ultrasound system (My Lab60; Esaote, Genova, Italy; probe: linear array LA523, 13 MHz, depth: 3.0 cm) was placed over the patellar tendon parallel to its longitudinal axis below the most distal apex of the patella and the central aspect of the tibial tuberosity, respectively.

**FIGURE 2:**
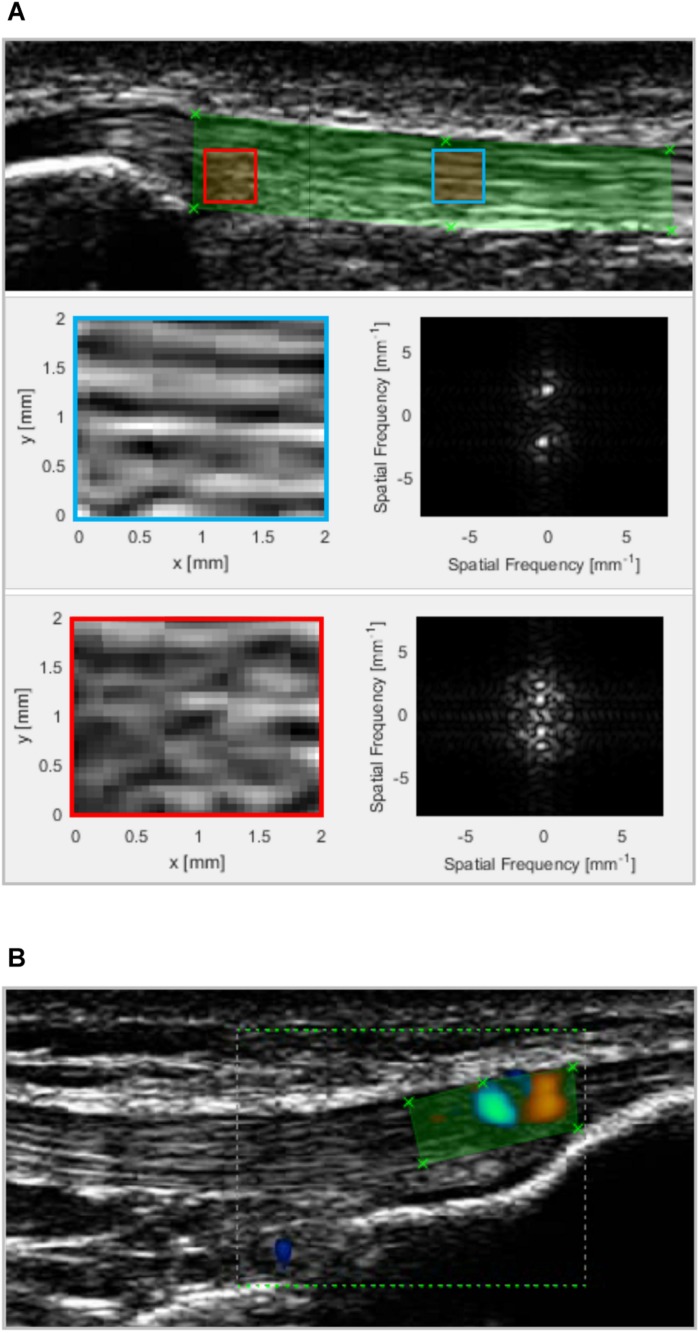
**(A)** Patellar tendon micromorphology was assessed by applying a spatial frequency analysis on ultrasound images obtained from the proximal (shown) and distal tendon. All possible 32 × 32 pixel kernels within a polygonal region of interest (ROI; green) were filtered and 2D fast Fourier transformed. The blue and red squares represent two kernels with a high and low degree of fascicle packing and alignment and, thus, peak spatial frequency (2.2 and 1.4 mm^–1^, respectively). The panels on the right to the enlarged kernels show the respective frequency spectrum. The average PSF value of all kernels was used in the statistical analysis. **(B)** Vascularisation was quantified by measuring the color area within a polygonal ROI that covered all intratendinous color doppler signals (exemplary distal image shown).

In the analysis of the captured images a polygonal region of interest (ROI) was defined in a custom written MATLAB interface (version R2016a; MathWorks, Natick, MA, United States). The length of the ROI corresponded to 40% of the tendon rest length of each participant (measured as described below) and its height covering the full thickness of the tendon. The location of the ROI was chosen that it spanned from the deep insertion to the central portion of the tendon, respectively. Within this ROI, as many kernels of 32 × 32 pixels as possible were analyzed as suggested by Bashford et al. (2008), by applying a 2D fast Fourier transform (FFT) and highpass filter with a radial frequency response and half-power cutoff frequency of 1.23 mm^–1^. The filtered kernels were zero-padded in both directions to a size of 128 × 128 pixels and the distance of the peak spatial frequency (PSF) from the spectral origin in the frequency spectrum was used as measure of the packing density and alignment of the collagen bundles (Bashford et al., 2008). See Figure 2A for an illustration of the procedure.

### Assessment of Tendon Neovascularization

A color Doppler examination to determine intratendinous blood flow was performed with the participants laying supine with the dominant knee flexed to 30° (0° = full extension). The joint angle was chosen based on former recommendations (Giombini et al., 2013; Bode et al., 2017) to reduce slack but avoid any passive forces that might induce vascular compression. The ultrasound system, probe model and orientation were as described above, yet the system settings were set as follows: CFM mode, 13 MHz frequency, 750 Hz peak repetition frequency, 1.1 by 1.6 cm color box size (spanning from the deep insertion at the patella or tibial tuberosity to the central portion of the tendon, respectively). The color gain was adjusted individually just below noise level. The probe pressure was kept to a minimum to avoid vascular obliteration. The tendon was scanned carefully in its full width moving the probe stepwise medio-laterally while keeping its longitudinal axis aligned with the longitudinal axis of the tendon. A short video sequence (i.e., ∼2–3 s) was captured at the site with the approximate strongest doppler signal. In the analysis, a polygonial ROI representing the tendon body within the color box was selected in the custom written MATLAB interface and the color area of the video frames were calculated based on the sum of all colored pixels in the ROI (see also Figure 2B). The largest color area of the sequence was used in the statistical evaluation.

### Statistical Analysis

Normality of the data was analyzed using the Shapiro–Wilk test. In case of normality, correlations between the main outcome parameters were calculated using the Pearson correlation coefficient (*r*) or else using Spearman’s rho (ρ). All statistical tests were run in SPSS (version 25, IBM, Armonk, NY, United States) with the alpha level set to 0.05.

## Results

**FIGURE 3:**
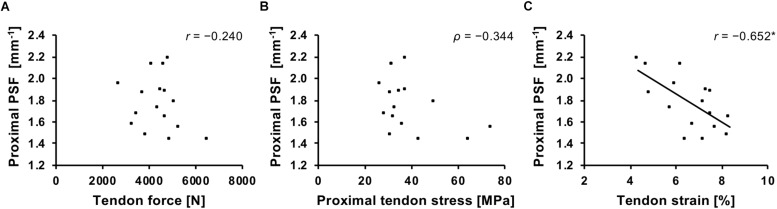
Association of patellar tendon force **(A)**, stress **(B)**, and strain **(C)** with the peak spatial frequency (PSF) of the proximal tendon. *r*, Pearson correlation coefficient; ρ, Spearman’s rho. ^*^p = 0.006.

The main outcome parameters of patellar tendon morphological and mechanical are presented in Table 1. Mean ± standard deviation of proximal and distal tendon PSF and color doppler area was 1.78 ± 0.25, 1.91 ± 0.13 mm^–1^ and 2.21 ± 4.11, 6.52 ± 5.89 mm^2^, respectively. The average VISA-P score of the affected participants (*n* = 6) was 73.8 ± 6.5 and for the unaffected 100 ± 0. There was a significant correlation of tendon strain and proximal tendon PSF (*r* = −0.652, *p* = 0.006; Figure 3). However, no significant association was found between strain and distal tendon PSF (*r* = 0.279, *p* = 0.3) or between tendon force (proximal: *r* = −0.24, *p* = 0.37; distal: *r* = −0.348, *p* = 0.19) or stress (proximal: ρ = −0.344, *p* = 0.19; distal: ρ = −0.068, *p* = 0.8) with tendon PSF at both sites, respectively (Figure 3). No significant correlations were observed between color doppler area and tendon mechanical parameters (ρ ranging from −0.17 to 0.08; *p* ≥ 0.54).

**TABLE 1:** Descriptive statistics of the patellar tendon morphological and mechanical properties of the investigated adolescent basketball athletes (*n* = 16).

	**Mean ± SD**
Tendon moment arm [mm]	54.1 ± 3.7
Tendon rest length [mm]	52.9 ± 6.8
Proximal tendon CSA [cm^2^]	1.18 ± 0.20
Distal tendon CSA [cm^2^]	1.19 ± 0.21
Tendon force [N]	4399 ± 898
Tendon stiffness [N/mm]	1338 ± 422
Normalized tendon stiffness [kN/strain]	73.1 ± 22.0
Proximal tendon stress [MPa]	38.7 ± 13.2
Distal tendon stress [MPa]	38.6 ± 13.3
Tendon strain [%]	6.6 ± 1.2

**CSA*: cross-sectional area.*

**FIGURE 4:**
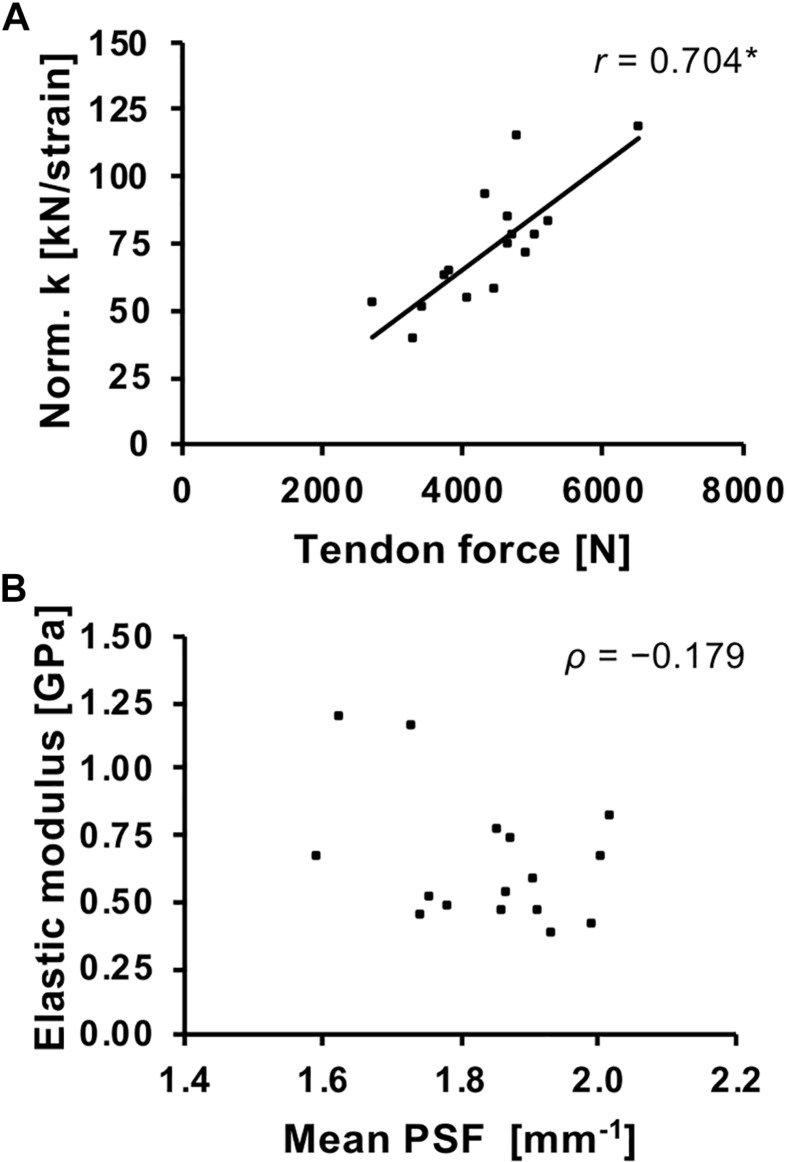
Association of patellar tendon force and normalized tendon stiffness (Norm. k; **A**) and average tendon PSF and elastic modulus **(B)**. *r*, Pearson correlation coefficient; ρ, Spearman’s rho. ^*^p < 0.01.

Normalized tendon stiffness correlated significantly with tendon force (*r* = 0.704, *p* = 0.002) but no significant correlation was found for tendon elastic modulus and average tendon PSF (ρ = −0.179, *p* = 0.506; Figure 4).

## Discussion

The present study investigated the association of patellar tendon loading (in terms of force, stress, and strain) and structural characteristics of the tendon in adolescent basketball athletes. We found a significant inverse correlation between tendon strain and proximal tendon PSF, suggesting that tendons that are subjected to a higher mechanical demand show lower levels of collagen fascicle organization. This was not found at the distal tendon site and no significant correlations were observed between tendon force or stress and tendon PSF as well as between tendon loading and vascularity. Finally, normalized tendon stiffness was closely associated to tendon force, yet, against our assumptions, the tendon elastic modulus was not associated with the measure of micromorphology (i.e., PSF). Therefore, our hypotheses were only partly confirmed.

Tendons commonly adapt to the force generating capacity of their associated muscles to maintain tendon strain during loading in a physiological range (LaCroix et al., 2013). Therefore, a close association of tendon force and stiffness has been reported earlier (Scott and Loeb, 1995; Arampatzis et al., 2007b; Waugh et al., 2011; Mersmann et al., 2016) and was confirmed for adolescent basketball athletes in the present study. However, within the course of a training process, imbalances in the adaptation of muscle and tendon can cause episodes of increased tendon strain (Mersmann et al., 2016). In the present study, the average tendon strain at 80% of the maximum tendon force was 6.6%, which approximates to 7.9% (ranging from 5.2 to 9.6%) for a maximum muscle contraction when extrapolated based on tendon stiffness. This compares well to what we observed earlier in adolescent volleyball athletes (Mersmann et al., 2016, 2017b), which demonstrated significantly higher levels of strain (7.6–8.0%) compared to similar-aged controls (5.5–6.4%). Repeated exposure to high levels of tendon strain might harm the integrity of the tendinous tissue and increase the risk of injury (Wren et al., 2003; Fung et al., 2009). In the present study, we found a significant inverse relationship of the patellar tendon strain during isometric contractions and the proximal tendon PSF, which estimates the level of organization of the collagenous scaffold. Low values of PSF correspond to a less compacted and more isotropic speckle pattern of the ultrasound images, which is characteristic for tendinopathic tendons (Bashford et al., 2008), likely due to increased water content and collagen disorganization (Xu and Murrell, 2008; Magnusson et al., 2010). Therefore, our data can be interpreted as evidence for structural degeneration in the patellar tendons of those adolescent basketball athletes that are subjected to high levels of strain, while peak force or stress show no direct association with structural degeneration.

**FIGURE 5:**
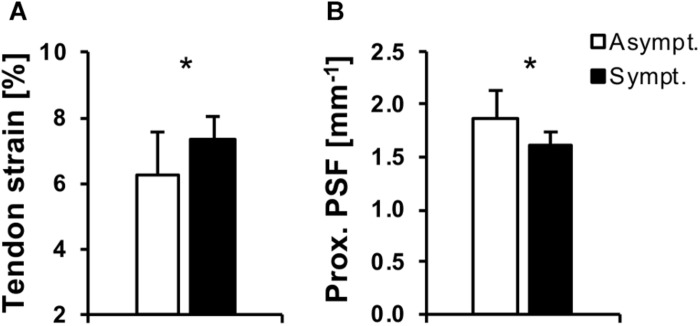
Patellar tendon strain **(A)** and proximal tendon peak spatial frequency (PSF; **B**) in adolescent basketball athletes that were asymptomatic or recovering from tendinopathy (white) or had persistent symptoms or developed tendinopathy (black). ^*^p < 0.05.

Indications for tendon matrix disorganization were present only at the proximal tendon site, which is also the most common site of pain and histological abnormalities (Roels et al., 1978; Ferretti et al., 1983). Lavagnino et al. (2008) further predicted greatest localized tendon strains at this region in a finite element model and found fascicle disruption at this location in their model-validation with cadaveric specimen. The finding is also in line with a cross-sectional study reporting lower proximal PSF values in symptomatic athletes compared to asymptomatic athletes and untrained controls, with no differences at the distal tendon (Kulig et al., 2013). In our study, we also assessed tendon pain at the time of data acquisition and again 2 month later using the VISA-P questionnaire. When clustering the participants of the present study to a group of asymptomatic athletes (reporting either no pain or recovering from tendinopathy to a VISA-P score of ≥ 85 after 2 months; *n* = 10) or symptomatic athletes (reporting pain on both occasions or developing tendinopathy within the 2 months; *n* = 6), we found significantly higher strain and lower proximal tendon PSF in the symptomatic group (Welch’s *t*-test *p* = 0.04 and 0.02, respectively; Figure 5). The two recovering participants that were reporting pain only in the first session and were included in the asymptomatic group did not show any indications of structural degeneration (PSF values of 1.87 and 1.88 mm^–1^, respectively), which suggests that pain in tendinopathy can persist or occur irrespective of tissue damage as determined by means of ultrasound. Though these results might need confirmation using a larger sample, taken together, these findings provide a strong argument for the assumption that high tendon strain induced by an imbalance of muscle strength and tendon stiffness associates to structural degeneration of the tissue at the proximal patellar tendon and increases the risk for tendinopathy in adolescent athletes.

In our study, we did not find a significant correlation between average tendon PSF and elastic modulus, suggesting that the material properties of the tendon are not directly linked to the structural organization as quantified by the PSF. This was against our assumptions, since – though moderate in magnitude – such an association has been reported earlier in the Achilles tendon (Kulig et al., 2016). However, there is a remarkable variability of tendon material properties between and within species (LaCroix et al., 2013) and the elastic modulus is strongly influenced by the type and degree of intra- and inter-fibrillar cross-linking (Thompson and Czernuszka, 1995; Depalle et al., 2015; Lin and Gu, 2015), glycosaminoglycan content (Cribb and Scott, 1995) and collagen area fraction (Robinson et al., 2004). Therefore, it is well-comprehensible that the variation in material properties cannot be simply explained by the alignment of collagen fascicles. The discrepancy to the associations reported earlier by Kulig et al. (2016) might be partly be related to the use of a single cross-section obtained at the thickest part of the tendon in that study, which results in an underestimation of elastic modulus for the whole tendon, especially in individuals that show localized tendon swelling (and, thus, a low PSF). However, it might also be that the findings are specific to the tendon investigated (patellar vs. Achilles) and it might still be possible that changes in the structural organization as quantified by means of a PSF analysis of ultrasound images might associate not to baseline material properties but to changes of those due to pathogenesis or repair. In other words, a change in tendon PSF could reflect changes in the material properties, though the absolute elastic modulus might not be predicted only based on the structural appearance.

We did not find an association of tendon loading parameters and neovascularization. Further, the frequency of doppler findings was higher in the distal part of the tendon (75%) compared to the proximal site (31%), though degenerative processes are more common at the latter and also pain was exclusively reported to concern the proximal tendon in the symptomatic athletes. Intratendinous Doppler flow has often been linked to the presence or development of tendinopathy (McAuliffe et al., 2016). However, this view has also been challenged (Tol et al., 2012), since Doppler signals are actually quite common in asymptomatic athletes (Gisslén and Alfredson, 2005; Gisslèn et al., 2005; Boesen et al., 2006; Sengkerij et al., 2009). Cyclic loading of tendons stimulates the expression of endothelial growth factors, which mediate blood flow and the formation and ingrowth of new vessels, in a frequency-dependent manner (Petersen et al., 2004). Dynamic mechanical loading further seems to be able to induce a persistent increase of intratendinous blood volume (Kubo et al., 2009). Therefore, it has been speculated that in elite-level athletes the presence of Doppler flow in tendons is actually a physiological response associated to the high frequency of training (Malliaras et al., 2008; Koenig et al., 2010) and, thus, may not be necessarily indicative for degenerative processes or related to the level force, stress, or strain imposed on the tendon.

Considering the age of the athletes participating in the present study, individual differences in biological maturity might have influenced the structural appearance of the tendon. Rudavsky et al. (2017) reported a high variability of the echo patterns of the patellar tendons of young athletes before and around PHV and more consistent and continuous patterns similar to mature tendons after 1 year post-PHV. Such maturation-related structural changes would also affect the estimation of micromorphology as applied in the present study. The estimated PHV-offset of the athletes in the present study was 0.9 ± 0.7 years post-PHV, with half of the individuals to be characterized as peri-PHV (PHV-offset between −1 and +1 year) and the other post-PHV (offset ≥ 1 year). Though the more mature participants demonstrated higher tendon forces and elastic modulus (*r* = 0.751 and ρ = 0.547, respectively; *p* < 0.05), using PHV-offset as controlling variable in a partial correlation between tendon strain and proximal PSF did not change the main outcome (*r* = −0.62; *p* = 0.014). Therefore, we are confident that mechanical strain was the main determinant for the microstructural characteristics of the patellar tendon in our sample.

A limitation of the present study is the lack of a comparison to an untrained group and, to some extent, the combination of both healthy and affected athletes. Therefore, our conclusions regarding the association of tendon strain and microstructure remain confined to adolescent athletes, though it seems unlikely that such an association could be established in untrained adolescents, due to the quite consistent tendon strain during maximum contractions observed in earlier studies (Mersmann et al., 2016). Yet it seems promising to explicitly investigate the development of tendon mechanical and structural characteristics in groups of symptomatic and asymptomatic athletes to gain further insight into the etiology of tendinopathy.

## Conclusion

The present study provides evidence that – in contrast to force or stress – high levels of tendon strain are associated to a micromorphological deterioration of the collagenous network in the proximal patellar tendon of adolescent basketball athletes. Further, athletes suffering from or developing tendinopathy demonstrated both greater levels of tendon strain and lower levels of fascicle packing and alignment, which lends support to the idea that mechanical strain is the primary mechanical factor for tendon damage accumulation and the progression of overuse (Schechtman and Bader, 1997; Wren et al., 2003; Fung et al., 2009). Though still challenging from a methodological perspective (Seynnes et al., 2015; Mersmann et al., 2018), monitoring tendon strain in athletes might in perspective be a promising approach to assess tendon injury risk and then prescribe exercises targeting an increase in tendon stiffness for individuals that have an imbalance of muscle strength and tendon stiffness (Mersmann et al., 2017a). The assessment of tendon micromorphology might additionally be used to estimate the structural development of the tendon, with an analysis of the proximal tendon probably being sensitive for discriminating healthy and affected tissue. Tendon vascularity on the other hand seems unrelated to the mechanical demand and could be a physiological response to frequent training in adolescent elite athletes.

## Data Availability

The datasets generated for this study are available on request to the corresponding author.

## Ethics Statement

The participants and legal guardians gave written informed consent to the experimental procedures, which were approved by the ethics committee of the Humboldt-Universität zu Berlin (Ethikkommission der Kultur-, Sozial-, und Bildungswissenschaftlichen Fakultät) and carried out in accordance with the declaration of Helsinki.

## Author Contributions

FM and AA conceived the experiments, interpreted the data, and drafted the manuscript. FM, M-ST, and NP performed the experiments. FM and M-ST analyzed the data. AS and AA substantially contributed to data analysis. M-ST, NP, and AS made important intellectual contributions during revision. All authors approved the final version of the manuscript and agreed to be accountable for the content of the work.

## Conflict of Interest Statement

The authors declare that the research was conducted in the absence of any commercial or financial relationships that could be construed as a potential conflict of interest.
